# Regulation of Adherens Junction Dynamics by Phosphorylation Switches

**DOI:** 10.1155/2012/125295

**Published:** 2012-07-12

**Authors:** Cristina Bertocchi, Megha Vaman Rao, Ronen Zaidel-Bar

**Affiliations:** ^1^Mechanobiology Institute Singapore, National University of Singapore, Singapore 117411; ^2^Department of Bioengineering, Faculty of Engineering, National University of Singapore, Singapore 119077

## Abstract

Adherens junctions connect the actin cytoskeleton of neighboring cells through transmembrane cadherin receptors and a network of adaptor proteins. The interactions between these adaptors and cadherin as well as the activity of actin regulators localized to adherens junctions are tightly controlled to facilitate cell junction assembly or disassembly in response to changes in external or internal forces and/or signaling. Phosphorylation of tyrosine, serine, or threonine residues acts as a switch on the majority of adherens junction proteins, turning “on” or “off” their interactions with other proteins and/or their enzymatic activity. Here, we provide an overview of the kinases and phosphatases regulating phosphorylation of adherens junction proteins and bring examples of phosphorylation events leading to the assembly or disassembly of adherens junctions, highlighting the important role of phosphorylation switches in regulating their dynamics.

## 1. Introduction

Adherens junctions (AJs) are cell-cell adhesion sites where calcium-dependent cadherin receptors bind with their extracellular domains to cadherins on opposing cells and with their cytoplasmic tails connect—via adaptors—to filamentous actin [1, 2]. By essentially providing a physical link between the actin cytoskeleton of neighboring cells AJs facilitate the integration of individual cells into a tissue. Additionally, AJs are instrumental in setting up and maintaining the apicobasal polarity of epithelial cells [3, 4], they function as mechanosensors [5] and serve as a nexus for signaling affecting important cell decisions, such as survival and differentiation [6].

During the development and lifetime of an organism, cells frequently change shape and position relative to their neighbors. Hence, the ability of cells to regulate their adhesive interactions plays a key role during tissue morphogenesis, repair, and renewal [3, 7, 8]. Defects in the adhesive characteristics of epithelial cells are pathological signs and loss of cell-cell adhesion can generate dedifferentiation and invasiveness of human carcinoma cells [9]. Thus, there is great interest in understanding the factors that affect assembly and disassembly of cell-cell adhesion at the molecular level.

When considering regulatory mechanisms controlling AJ proteins, we distinguish between three subsequent steps of regulation: synthesis, localization, and activation. First, a cell controls whether proteins are synthesized or not. Indeed, transcriptional regulation of E-cadherin, notably by the snail transcription factor, plays an important role in the breaking down of AJs accompanying epithelial to mesenchymal transition [10]. Once a protein is expressed the cell can determine its localization by controlling its transport. In fact, both exocytosis and endocytosis of E-cadherin are tightly controlled and the balance between the two processes has been shown to regulate AJ turnover both *in vitro* and *in vivo* [6]. Finally, a cell can control the activity and interactions of a protein at a given location by posttranslational modifications. These modifications include glycosylation, lipidation, ubiquitination, acetylation, proteolysis, and phosphorylation [11]. Phosphorylation of tyrosine (Y), serine (S), or threonine (T) residues, the topic of this review, is a rapid and reversible form of regulation affecting the majority of AJ proteins [12–16]. In some cases, posttranslational modifications have secondary effects on transcription and/or protein transport [17, 18]. However, here we will focus on the more direct mechanisms in which AJs are regulated by phosphorylation. First, we will introduce the enzymes responsible for phosphorylation and dephosphorylation at AJs and discuss how they are recruited into AJ and activated. Next, we will describe the targets of phosphorylation within AJ and by examining the consequences of specific phosphorylation events will show how phosphorylation is involved both in assembly and disassembly of AJ, essentially driving the dynamics of this highly responsive structure. In the end, we will point out open questions and suggest methods to address them.

## 2. Recruitment of Protein Kinases and Phosphatases into AJ

So far, twelve S/T kinases and one S/T phosphatase have been implicated in regulating phosphorylation of AJ proteins, and they are all cytoplasmic (Table 1). Prominent kinases in this group include PKC-*α*, cAMP-dependent protein kinase, Casein Kinase 1, Pak1, and ROCK1. Nine tyrosine kinases and twelve tyrosine phosphatases have been shown to be active in AJ, roughly half of them cytoplasmic and half part of a transmembrane receptor (Table 1). Prominent tyrosine kinases include the cytoplasmic Src, Fyn, Fer, and Abl, and the receptors of epidermal and hepatocyte growth factors. Major phosphatases involved are the cytoplasmic PTP-1B, PTP-PEST, SHP-1, SHP-2, and receptor-type tyrosine-protein phosphatases Mu, U, and Kappa.

Some of these kinases and phosphatases have been localized to AJ by immunofluorescence (e.g., [19–21]) and others have been shown to associate with AJ by coimmunoprecipitation (e.g., [21–23]), but the exact mechanism of recruitment into AJ of most of them is largely unknown. A few were shown to bind directly with cadherin, such as CSK with VE-cadherin and PTP-1B with E- and N-cadherin [19, 24, 25]; others bind one of the catenins (adaptor proteins linking cadherin with actin), such as MET and PTPRF with *β*-catenin [26, 27] and ROCK1 with p120-catenin [21]; some interact with other AJ adaptor proteins, such as PRKCA with vinculin and ROCK1 with Shroom3 [28, 29].

While it is most likely every kinase and phosphatase can recognize at least one docking site within the AJ, it is not currently known which of the kinases and phosphatases reside in AJ permanently and which are transient components, homing in to phosphorylate or dephosphorylate AJ proteins only under specific conditions. Even permanent residents may not always be active, as most kinases and phosphatases need themselves to be activated.

## 3. Activation of Kinases and Phosphatases in AJ

**Figure 1:**
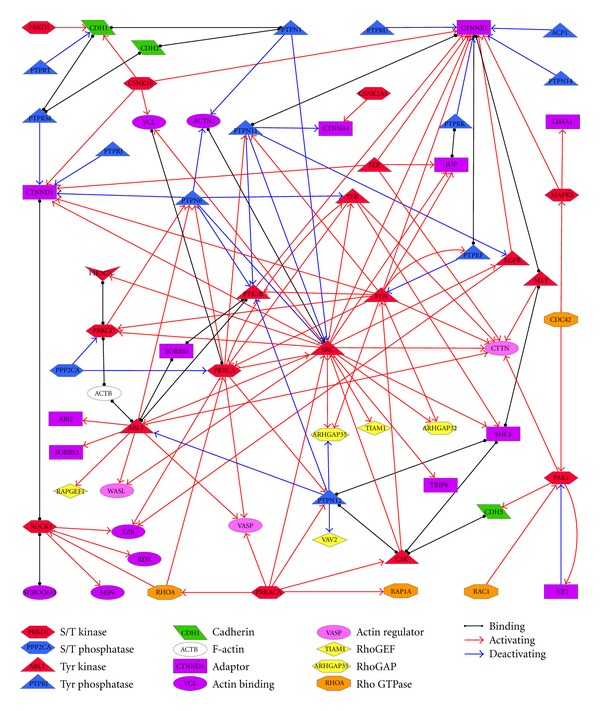
Network of phosphorylation enzymes and targets in the adherens junction.

Receptor tyrosine kinases are commonly activated by an external ligand, such as a growth factor or cytokine, which induces dimerization, cis-phosphorylation or autophosphorylation and activation of the catalytic domain [124, 125]. Receptor tyrosine phosphatases may be activated by homophilic association with their counterparts on neighboring cells [126], as well as by tyrosine phosphorylation [127]. Several S/T kinases are activated by binding of Rho GTPases, for example ROCK1 is activated by RhoA and PAK1 is activated by Rac1 and Cdc42 [128, 129]. S/T kinases are also regulated by tyrosine phosphorylation and tyrosine kinases and phosphatases are regulated by S/T phosphorylation, in a complex web of feedback and feedforward loops that is poorly understood (Figure 1). For example, Src phosphorylates PRKCD, which phosphorylates PTPN6, which in turn dephosphorylates SRC (feedback) [130–132]; PRKACA phosphorylates Src and Csk, and Csk also phosphorylates Src (feedforward) [133–135].

As will be discussed further below, some of the phosphorylation events serve to activate the kinases or phosphatases and others are inhibitory. One well-understood example of kinase activation is the mechanism of activation of Src. As reviewed in [136], the family of Src tyrosine kinases can be found in a nonactive “closed” conformation or in an “open” active conformation, depending on the phosphorylation status of a tyrosine residue at the C-terminus. When this residue is phosphorylated, it interacts with an SH2 domain in the middle of Src, blocking the catalytic site. Upon dephosphorylation of this specific tyrosine, the SH2 domain is released, and the protein unfolds, allowing autophosphorylation of another tyrosine residue situated within the enzyme's activation loop, rendering the kinase fully active [137]. It is important to point out that cadherin ligation and clustering may act as an activation signal for some kinases. Most notably, Src and Fer have been shown to be recruited to the membrane upon cadherin binding [138, 139], and EGFR signaling was shown to be stimulated by AJ formation independently of EGF ligand [140]. Furthermore, cadherin clustering has been found to indirectly induce activation of Rho GTPases [141], which in turn could activate S/T kinases.

## 4. Phosphorylation Targets within the AJ

The AJ can conceptually be divided into four layers (Table 2). The first, in the plane of the membrane, is where cadherins and other transmembrane proteins, such as nectin and AJAP1, reside. The next layer consists of membrane-bound adaptors, such as ERM proteins and MAGI1, and adaptors that directly bind transmembrane proteins, such as p120- and *β*-catenin (bind cadherin) and afadin (binds nectin). The following layer is composed of adaptor proteins, such as *α*-catenin and vinculin, which bind to the second layer adaptors and also bind F-actin. F-actin, along with actin-binding proteins, such as *α*-actinin, and actin regulators, such as cortactin, would be considered the last layer. Regulatory proteins, such as GAPs, GEFs, and GTPases, can be found throughout the AJ as reviewed in [14, 142].

There is evidence demonstrating both Y and S/T phosphorylation of proteins in all layers of the AJ (Table 2). As illustrated in Figure 1, often the same kinase will phosphorylate proteins from different layers. For example, Abl phosphorylates actin regulators WASP and VASP [143, 144], as well as cadherin-bound adaptor *δ*-catenin [145] and second layer adaptors Abi2 and Vinexin (SORBS3) [146, 147].

Some AJ proteins have additional functions in the cell, and phosphorylation is also involved in regulating their non-AJ roles [148]. The most notable example is *β*-catenin, which plays an important role in the Wnt signaling pathway as a cotranscription factor of TCF/LEF [149]. Whether nonjunctional *β*-catenin will reach the nucleus or not depends on whether it is phosphorylated by GSK3 and Casein Kinse I in the “destruction complex” [150]. However, such phosphorylation events taking place outside the context of AJ are beyond the scope of this paper.

For the phosphorylation events occurring within AJ an important question is how do they affect the target proteins?

## 5. Consequences of Phosphorylation on AJ Proteins

A phosphorylated tyrosine, serine, or threonine residue can affect a protein in three major ways: it can increase the affinity for another protein, it can inhibit a protein-protein interaction, or it can activate enzymatic activity. In proteins with an intramolecular interaction, phosphorylation and dephosphorylation can elicit a conformational change in the protein. AJ components provide examples of each type of these outcomes, as detailed henceforth.

**(1) Turn “on” Protein-Protein Interaction**

Tyrosine phosphorylation can create a docking site for an SH2 or PTB domain of a partner protein. For example, tyrosine phosphorylation of cadherin creates docking sites for the SH2 domain of the adaptor SHC1 [151] and the PTB domain of the cell polarity protein Numb [152]. As mentioned earlier, SRC family kinases are inhibited by an intramolecular interaction between a central SH2 domain and a phosphorylated tyrosine at the C-terminus [137].

**(2) Turn “off” Protein-Protein Interaction**

Examples of interaction inhibition by phosphorylation are also found both between different proteins and intramolecularly: tyrosine phosphorylation of VE-cadherin at certain residues prevents the binding of p120-catenin and *β*-catenin [153]; phosphorylation of a threonine residue in the C-terminal actin binding domain of ERM proteins interferes with its interaction with the N-terminal FERM domain, helping to keep the protein in an active open conformation [154].

**(3) Turn “on” Enzymatic Activity**

Activation of the catalytic activity of tyrosine kinases and phosphatases by tyrosine phosphorylation has already been mentioned above [127]. Another important example is the activation of the motor activity of myosin by the phosphorylation of serine and threonine residues of myosin light chain [155].

We next address the question what are the ramifications of phosphorylation of AJ proteins on AJ structure and dynamics.

## 6. Global versus Specific Consequences of Phosphorylation on AJ Structure and Dynamics

Numerous experiments have been carried out over the years to address the role of Y and S/T phosphorylation in regulating AJ. Early experiments used broad-spectrum chemical inhibitors of kinases or phosphatases to conclude that phosphorylation negatively impacts cadherin function. For example, inhibition of S/T phosphatases by Okadaic acid or Calyculin-A was reported to lead to complete disassembly of AJ within an hour, and this disruption was attributed to an increase in S/T phosphorylation of *β*-catenin [89]. However, Calyculin-A has also been shown to increase actomyosin contractility in cells [156], suggesting that the disruption of AJ in the above mentioned study may have been caused by mechanical tension at the junctions exceeding their adhesive strength. Inhibition of tyrosine phosphatases with sodium orthovanadate was reported to lead within minutes to a dramatic increase in phosphotyrosine signals at AJ, followed by the disassembly of AJ [157]. Consistent with the notion that excessive tyrosine phosphorylation in AJ causes their disassembly, cells expressing constitutively active Src kinase lost their AJ, and inhibition of tyrosine kinase activity by the drug tyrphostin was able to restore AJ in the Src-transformed cells [157]. These and similar experiments have led researchers in the late 90s of the previous century to the general conclusion that phosphorylation is a negative regulator of AJ.

However, in more recent years, there is accumulating evidence for a positive role of phosphorylation in AJ assembly, mainly coming out of loss-of-function experiments of specific kinases. For example, SRC and FYN were found to be essential for the formation of AJ in mouse keratinocytes [158]. Moreover, SRC activity was shown *in vitro* to be important for the recruitment of PI3K to AJ and the ability of cells to expand nascent cadherin-adhesive contacts [159]. Along the same lines, ABL1 tyrosine kinase activity was shown to be important for the maintenance of adherens junctions in epithelial cells [37], and S/T phosphorylation of E-cadherin by protein kinase D1 (PRKD1) was found to be associated with increased cellular adhesion and decreased cellular motility in prostate cancer [59].

Hence, the emerging view is that it is not possible to generalize the effect of phosphorylation on AJ. With some phosphorylation events leading to the switching “on” of a protein or interaction and other phosphorylation events, even on the same protein, serving as a switch “off”, the effect of phosphorylation on AJ dynamics has to be examined on a residue-by-residue basis. After we delineate the effect of each individual phosphorylation event, we should be able to integrate this information into a single network of interconnected switches and perhaps then we can follow the global effects of a single phosphorylation switch, starting, for example, with hepatocyte growth factor stimulation [160].

## 7. Consequences of Specific Phosphorylation Events on AJ, Composition and Dynamics

We close this paper by giving a few examples of cases in which the consequences of specific phosphorylation events are known. The phosphorylation events presented occur on proteins from each layer of the AJ as well as one cell polarity protein.

**(1) Cadherin**

Serine phosphorylation of residues S840, S851 and S853 in the C-terminus of human E-cadherin (likely by CSNK1E or PRKD1) increases the binding affinity towards *β*-catenin, whereas phosphorylation of S846 is said to inhibit the same interaction [86]. Stronger binding of *β*-catenin to E-cadherin is conducive to a stronger AJ structure. Tyrosine phosphorylation of VE-cadherin at two critical tyrosines, Y658 and Y731, is sufficient to prevent the binding of p120- and *β*-catenin, respectively [161]. Phosphorylation by Src of three tyrosines in position 753–755 on human E-cadherin creates a docking site for the E3-ligase Hakai [162]. Ubiquitination of E-cadherin by Hakai leads to internalization of E-cadherin facilitating disassembly of the AJ [162, 163].

**(2) P120-Catenin**

Eight tyrosine residues in the N-terminus of p120-catenin can be phosphorylated by Src [94]. Upon phosphorylation, these sites serve as docking sites for the recruitment of interacting proteins carrying SH2 domains, such as the tyrosine phosphatase SHP-1 [164]. Under certain conditions tyrosine phosphorylation of p120-catenin was shown to increase its affinity to cadherin, while in other instances such an increase was not observed (reviewed in [95]). The affinity of p120-catenin to cadherin is significant for AJ dynamics because p120-catenin protects cadherin from being internalized [165].

**(3) Zyxin**

Phosphorylation of S142 of zyxin is thought to result in the release of an intramolecular head-tail interaction [166]. Opening of the protein expose its ACTA repeats that recruit VASP, whose actin polymerization activity (see below) is important for AJ assembly and maintenance. Since zyxin-mediated recruitment of VASP has a positive effect on AJ [167, 168], it is not surprising that expression of a zyxin phosphomimetic mutant results in ultrastable AJ [166].

**(4) VASP (Vasodilator Stimulated Phosphoprotein)**

As its name suggests, VASP is often found phosphorylated in cells. Three phosphorylation sites on residues S157, S239, and T274 are phosphorylated by PKA and PKG, as well as PKC [169, 170] and dephosphorylated by unknown phosphatase/s. The phosphorylation of VASP was shown to reduce its affinity towards actin [171] and essentially turn off its actin bundling and anticapping/elongation activity [171, 172]. VASP-mediated actin elongation is important for the formation of AJ and for the maintenance of actin structures associated with AJ [173, 174]. Thus, the consequence of VASP phosphorylation is to negatively regulate AJ assembly and maintenance.

**(5) PARD3**

*In Drosophila* epithelial cells the par-3 ortholog Bazooka is confined to AJ as a result of phosphorylation by either apical or basal polarity complexes [4]. At the apical side of cells Bazooka is phosphorylated by aPKC, resulting in its release from the cortex [175, 176]. In the basolateral membrane Par1 kinase phosphorylates Bazooka on unique sites that also lead to its cortical release [4]. Recently, it was shown that the ratio between Par-1 and aPKC determines the position of Bazooka and AJ along the lateral side and a reduction in Par-1 kinase activity leads to a basal shift of AJ followed by folding of the epithelial sheet [177].

## 8. Conclusions and Outlook

From the examples presented above, it is clear that phosphorylation switches play a pivotal role in regulating AJ assembly and disassembly dynamics. At the same time it is also clear that our knowledge is only scratching the surface of the phosphorylation network regulating AJ. For the majority of known phosphorylation events in AJ, we know either of a kinase or of a phosphatase involved, but rarely do we know both. Furthermore, while traditional biochemistry techniques have facilitated the characterization of a handful of phosphorylation events on AJ proteins, phosphoproteomic data indicates that the majority of AJ proteins are phosphorylated on multiple serine/threonine and tyrosine residues [178]. Phosphoproteomics, which utilizes a variety of techniques to label cells, enrich for phosphorylated peptides and identify them using mass-spectrometry (reviewed in [179–181]), not only highlights the hole in our knowledge but also offers the means to fill it.

Phospho-proteomics offers an unbiased and comprehensive snapshot of phosphorylation events, and several different approaches can be taken to elucidate phosphorylation switches in AJ: during normal assembly and maturation, following a signal for disassembly, or when a certain kinase or phosphatase is activated or missing (e.g., [39, 182–184]). The phospho-proteomic data obtained, especially if it is dynamic, can be used for a systems level analysis of phosphorylation switches in AJ [185, 186], but it seems likely to us that before the network can be modeled in a meaningful way more in depth characterization of specific phosphorylation events will be necessary, using cell biological techniques.

While for the discovery and mapping of phosphorylation events in AJ, one wants to be as comprehensive as possible, when it comes to characterizing a particular switch the more specific the tools, the better. One example of a specific tool is phosphorylation site-specific antibodies, such as those recognizing individual phosphorylation events on *β*-catenin and p-120-catenin [187, 188]. Another example are site-specific phospho-mimetic or nonphosphorylatable mutations, such as those successfully applied to the study of the effects of phosphorylation on cortactin, VASP, VE-cadherin, zyxin, and paxillin [153, 166, 189–191].

Facing an ever-changing landscape of forces and signaling cues, a cell must respond rapidly by adjusting the strength of its AJs according to need. For this it relies on continuous turnover and assembly of core AJ components. Phosphorylation is particularly suitable for regulating the balance between assembly and disassembly as it is rapid and affects the AJ proteins directly. Feedback loops must guarantee a combination of phosphorylated residues at AJ that matches the requirements for a given condition. Experiments have shown that when it comes to phosphorylation both “all on” and “all off” treatments are deleterious to AJ. The challenge now is to elucidate the mechanisms by which the cell maintains a “just right” level of phosphorylation in AJ. While phosphorylation is probably the most prominent regulatory switch controlling cell adhesion, other switches, such as GTPases, lipids and proteases, do exist [192]. A future challenge, therefore, will be to integrate the phosphorylation switch network with the other regulatory switches to facilitate a true understanding of how different signaling pathways and force regulate AJ dynamics.

## Figures and Tables

**Table 1:** Kinases and phosphatases regulating phosphorylation of AJ proteins.

Gene symbol	Protein name	Phosphorylation type	Localization	Reference
*Kinases *				
SRC	Proto-oncogene tyrosine-	Tyr	nonreceptor	[23, 30, 31]
	protein kinase Src			
CSK	c-src tyrosine kinase	Tyr	nonreceptor	[24, 32, 33]
FYN	Tyrosine-protein kinase Fyn	Tyr	nonreceptor	[34–36]
ABL1	Abl1	Tyr	nonreceptor	[37, 38]
SYK	Tyrosine protein kinase SYK	Tyr	nonreceptor	[39, 40]
PTK2B	Protein-tyrosine kinase 2-beta	Tyr	nonreceptor	[41–43]
FER	Tyrosine-protein kinase Fer	Tyr	nonreceptor	[44]
EGFR	Epidermal growth factor receptor	Tyr	Receptor	[45]
cMET/HGF	Hepatocyte growth factor receptor	Tyr	Receptor	[46]
PRKCA	Protein kinase C alpha type	Ser/Thr	nonreceptor	[47]
PRKACA	cAMP-dependent protein	Ser/Thr	nonreceptor	[48, 49]
	Kinase catalytic subunit alpha			
ROCK1	Rho-associated, coiled-coil	Ser/Thr	nonreceptor	[50]
	containing protein kinase 1			
PRKCD	Protein kinase C delta type	Ser/Thr	nonreceptor	[51, 52]
CSNK1E	Casein kinase I isoform epsilon	Ser/Thr	nonreceptor	[53]
CSNK2A1	Casein kinase 2	Ser/Thr	nonreceptor	[54]
PAK1	Serine/threonine-protein kinase PAK 1	Ser/Thr	nonreceptor	[55–57]
MAPK8	JNK	Ser/Thr	nonreceptor	[58]
PRKD1	Protein kinase D1	Ser/Thr	nonreceptor	[59]
PRKCI	Atypical protein kinase C-lambda/iota	Ser/Thr	nonreceptor	[60]
PRKCZ	Protein kinase C zeta type	Ser/Thr	nonreceptor	[60]
MARK2	MAP/microtubule affinity-	Ser/Thr	nonreceptor	[61]
	Regulating kinase 2, Par-1			

*Phosphatases *				
PTPN1	Tyrosine-protein phosphatase non	Tyr	nonreceptor	[19, 62–64]
	receptor type 1, PTP1B			
PTPN6	Tyrosine-protein phosphatase non	Tyr	nonreceptor	[65]
	receptor type 6, SHP1			
PTPN11	Tyrosine-protein phosphatase non-	Tyr	nonreceptor	[66]
	receptor type 11, SHP2			
PTPN12	Tyrosine-protein phosphatase non-	Tyr	nonreceptor	[67]
	receptor type 12, PTP-PEST			
PTPN14	Tyrosine-protein phosphatase non-	Tyr	nonreceptor	[68]
	receptor type 14, PEZ			
ACP1	Acid phosphatase of erythrocyte, LMW-PTP	Tyr	nonreceptor	[69, 70]
PTPRJ	Receptor-type tyrosine-protein	Tyr	Receptor	[71]
	phosphatase eta (R-PTP-eta), DEP1			
PTPRM	Receptor-type tyrosine-protein	Tyr	Receptor	[72–74]
	phosphatase mu (RPTP mu)			
PTPRT	Receptor-type tyrosine-protein	Tyr	Receptor	[75]
	phosphatase T (R-PTP-T)			
PTPRU	Receptor-type tyrosine-protein	Tyr	Receptor	[76, 77]
	phosphatase U (R-PTP-U)			
PTPRK	Receptor-type tyrosine-protein	Tyr	Receptor	[78, 79]
	phosphatase kappa			
PTPRF	Receptor-type tyrosine-protein	Tyr	Receptor	[80–82]
	phosphatase F, LAR			
PPP2CA	Serine/threonine-protein phosphatase 2A	Ser/Thr	nonreceptor	[83–85]
	catalytic subunit alpha isoform			

**Table 2:** Targets of phosphorylation in AJ.

Gene symbol	Protein name	Phosphorylated residue	Reference
*Transmembrane*			
CDH1	Ecadherin	S/Y	[54, 86, 87]
PVRL1	Nectin	Y	[88]

*Cadherin- or membrane bound*			
CTNNB1	*β*-catenin	S/T/Y	[16, 89, 90]
EZR, RDX, MSN	ERM proteins (ezrin/radixin/moesin)	S/T/Y	[91–93]
CTNND1	p_120_-catenin	S/T/Y	[94, 95]
JUP	Gamma-catenin	Y	[96]
PARD3	Partitioning defective 3 homolog	S/T/Y	[97, 98]

*Secondary adaptors*			
CTNNA1	*α*-catenin	S/T/Y	[99, 100]
VCL	Vinculin	S/Y	[101, 102]
LIMA1	Eplin	S	[103]
VASP	Vasodilator-stimulated phosphoprotein	S/T/Y	[104–106]
SHC1	SHC-transforming protein 1	Y	[107, 108]

*Actin and actin regulators*			
ACTN1	*α*-actinin	S/Y	[109, 110]
CTTN	Cortactin	S/T/Y	[111, 112]
ACTB	F-actin	S/Y	[113–115]

*GTPASE regulators*			
PI3K	Phospho-inositide-3-kinase	Y	[116]
RAPGEF1	Rap guanine nucleotide exchange factor 1	Y	[117]
ARHGAP35	rho GAP p190A	Y	[118, 119]
ARHGAP32	p200RhoGAP	Y	[120, 121]
TIAM1	T-lymphoma invasion and metastasis-inducing protein 1	Y	[122]
VAV2	Vav 2 guanine nucleotide exchange factor	Y	[122, 123]
